# Carbon sequestration potential of different forest types in Pakistan and its role in regulating services for public health

**DOI:** 10.3389/fpubh.2022.1064586

**Published:** 2023-01-13

**Authors:** Shahab Ali, Shujaul Mulk Khan, Zeeshan Ahmad, Zafar Siddiq, Abd Ullah, Sunghoon Yoo, Heesup Han, António Raposo

**Affiliations:** ^1^Department of Plant Sciences, Quaid-i-Azam University, Islamabad, Pakistan; ^2^Member Pakistan Academy of Sciences, Islamabad, Pakistan; ^3^Department of Botany, Government College University, Lahore, Pakistan; ^4^Audit Team, Hanmoo Convention (Oakwood Premier), Seoul, Republic of Korea; ^5^College of Hospitality and Tourism Management, Sejong University, Seoul, Republic of Korea; ^6^CBIOS (Research Center for Biosciences and Health Technologies), Universidade Lusófona de Humanidades e Tecnologias, Lisboa, Portugal

**Keywords:** trees functional traits, carbon sequestration, elevation, structural equation model, regional scale, air quality

## Abstract

A high amount of CO_2_ causes numerous health effects, including headaches, restlessness, difficulty in breathing, increased heart rate, high blood pressure, asphyxia, and dizziness. This issue of increasing atmospheric CO_2_ can only be solved *via* above-ground and below-ground carbon sequestration (CS). This study was designed to determine the relationship between CS with the crown area (CA), diameter at breast height (DBH), height (H), species richness (SR), and elevation in different forest types of Pakistan with the following specific objectives: (1) to quantify the direct and indirect relationship of carbon sequestration with CA, DBH, H, and SR in various natural forest types and (2) to evaluate the effect of elevation on the trees functional traits and resultant CS. We used the linear structural equation model (SEM) for each conceptual model. Our results confirmed that the highest CS potential was recorded for dry temperate conifer forests (DTCF) i.e., 52.67%, followed by moist temperate mix forests (MTMF) and sub-tropical broad-leaved forests (STBLF). The SEM further described the carbon sequestration variation, i.e., 57, 32, 19, and 16% under the influence of CA (β = 0.90 and *P*-value < 0.001), H (β = 0.13 and *p*-value = 0.05), DBH (β = 0.07 and *p*-value = 0.005), and SR (β = −0.55 and *p*-value = 0.001), respectively. The individual direct effect of SR on carbon sequestration has been negative and significant. At the same time, the separate effect of CA, DBH, and H had a positive and significant effect on carbon sequestration. The remaining 20% of CS variations are indirectly influenced by elevation. This means that elevation affects carbon sequestration indirectly through CA, DBH, H, and SR, i.e., β = 0.133 and *P*-value < 0.166, followed by β = 0.531 and *P*-value < 0.001, β = 0.007 and *P*-value < 0.399, and β = −0.32 and *P*-value < 0.001, respectively. It is concluded that abiotic factors mainly determined carbon sequestration in forest ecosystems along with the elevation gradients in Pakistan. Quantifying the role of various forest types in carbon dioxide (CO_2_) reduction leads to improved air quality, which positively impacts human health. This is an imperative and novel study that links the dynamics of the biosphere and atmosphere.

## 1. Introduction

The forest ecosystem provides clean air through carbon sequestration for better public health. The sequestered carbon is not emitted into the atmosphere, reducing the chances of various human diseases (1). Forest ecosystems determine global climate change by removing or adding greenhouse gases such as CO_2_ from the atmosphere. Forests, alongside the grassland and peat swamps, collectively store more carbon than other terrestrial ecosystems (2). Forest trees store CO_2_ from the atmosphere in the form of wood, soil organic carbon, and other biomasses which contribute to a reduction in global warming and climate change (3). Disturbances in these ecosystems due to the overexploitation of resources cause the release of a significant amount of CO_2_ back into the atmosphere (4).

Carbon sequestration is essential to mitigate global climate change (5). Approximately 53% of carbon is stored in temperate and boreal regions, while the remaining 37 % is stored in tropical areas (6, 7). Half of the terrestrial carbon sink is located in forest ecosystems (8). Forest ecosystems take a large amount of CO_2_ from the atmosphere *via* photosynthesis. They also contribute a large amount of fixed carbon in the form of organic matter in the lithosphere (9). However, a small portion of integrated carbon has been stored in the belowground biomass, litter, and soil (10). According to United Nations Food and Agriculture Organization (FAO) statistics, forests contain 234 Pg of carbon in the above-ground forest, 62 Pg below ground, 41 Pg in the form of deadwood, 23 Pg in litter form, and 398 Pg in the forest soils (11). Forest ecosystems occupy the most significant part of the free-ice land surface in the entire terrestrial world. Trees are the main component of the forest ecosystem and contain the entire quantity of forest living biomass. Forests' total biomass is ~677 Petagram (PgC), and trees contribute about 80% of the world's total biomass (11).

Several biotic and abiotic factors influence carbon sequestration in forest ecosystems (12–14). Trees continuously sequester CO_2_ from the atmosphere and store it in their different parts, i.e., stem, root leaves, and branches (15). The carbon sequestration rate depends on plant growth, individual characteristics of the tree species, the wood density, and its growing conditions. Trees store most of the atmospheric carbon because of their larger size, volume, and long-lived storage capacities, i.e., tree trunks, leaves, roots, and the soils in which they exist (16). Carbon sequestration is mainly driven through huge tree biomasses (17).

The scientific community is committed to reducing atmospheric CO_2_ emissions and storing them in any other form to improve the quality of the atmosphere (18). Various biological and geological mechanisms bring free carbon down from the atmosphere. However, due to the enormous rise in human population, there is a continuous degradation of the land and deforestation, which results in the irreversible loss of forest functions. Plants are the primary producers of the world's biodiversity. They capture atmospheric CO_2_ and convert it into glucose, the first organic molecule of life (19). Degraded forests lead to low carbon storage and poor biodiversity, which in turn cause global warming and climatic changes (20). Anthropogenic activities drastically add massive amounts of greenhouse gases, mainly CO_2_, into the atmosphere (16). The rapid increase in the concentration of CO_2_, methane, and methane dioxide is the major cause of global warming (21).

Characteristically, above-ground biomass decreases along the elevation, but precipitation influences the biomass variations alongside the elevation gradient (22). Carbon sequestration is mainly determined by the biotic determinant of trees, such as variations in diameter, height diversity, stand density, and stand basal area (20, 23). The individual tree height is not associated with diameter, but the ratio is correlated to the species' genetic nature, intraspecific competition, and abiotic drivers like precipitation, temperature, and soil types (24–26). However, tree heights decline with increasing elevation, generally (27). Initially, researchers use DBH data to estimate carbon sequestration, but the precision of carbon estimation has improved using tree height data (28, 29). Proper forest administration and land improvement approaches that concurrently enhance the biodiversity and carbon stock to mitigate climate change and global warming across regional and global scales are required to bring positive changes at least at the micro-environment levels (30). However, the relationship between forest stand structure, i.e., height, diameter at breast height, crown area, and species richness along regional level elevation gradients in different forest types, have rarely been evaluated. Therefore, the current study was designed to quantify the carbon sequestration potential of various forest types in Pakistan, focusing on its biotic complexities.

### 1.1. Theoretical framework

The net primary productivity theory explains how forest productivity is affected by forest structure, i.e., First, the net immediate productivity drops during stand expansion due to the limited supply of water and nutrients to leaves within a forest stand. Second, how productivity changes systematically with climate change interaction and withstands developmental processes. These arguments provide predictions about variability in wood production, biomass loss rate, and carbon sequestration. The literature shows that the forest ecosystem is driven by biotic and abiotic factors such as forest age, forest origin, forest soil conditions, and geography of the region. Forest structure and growth are controlled by vegetation cover. Through aerial expansion, trees increase their potential height, producing more biomass (31, 32). The effects of abiotic and biotic factors on carbon sequestration at large regional-scale forest ecosystems have rarely been studied. Thus, the driving biotic and abiotic factors of carbon sequestration in multiple forest types are vague and need to be explored.

Stand structure is associated with differences in species composition, and there is a positive relationship between functional and taxonomic species diversity with the above-ground biomass (23, 33). The relationship between carbon sequestration with tree crown area, diameter at breast height, height, elevation, and species richness is usually positive. Still, the association and simultaneous direct and indirect impacts in different forest ecosystems remain questionable. Therefore, current studies were conducted in diverse forest ecosystems to evaluate the role of various biotic and abiotic factors on carbon sequestration at a regional level.

**Figure 1:**
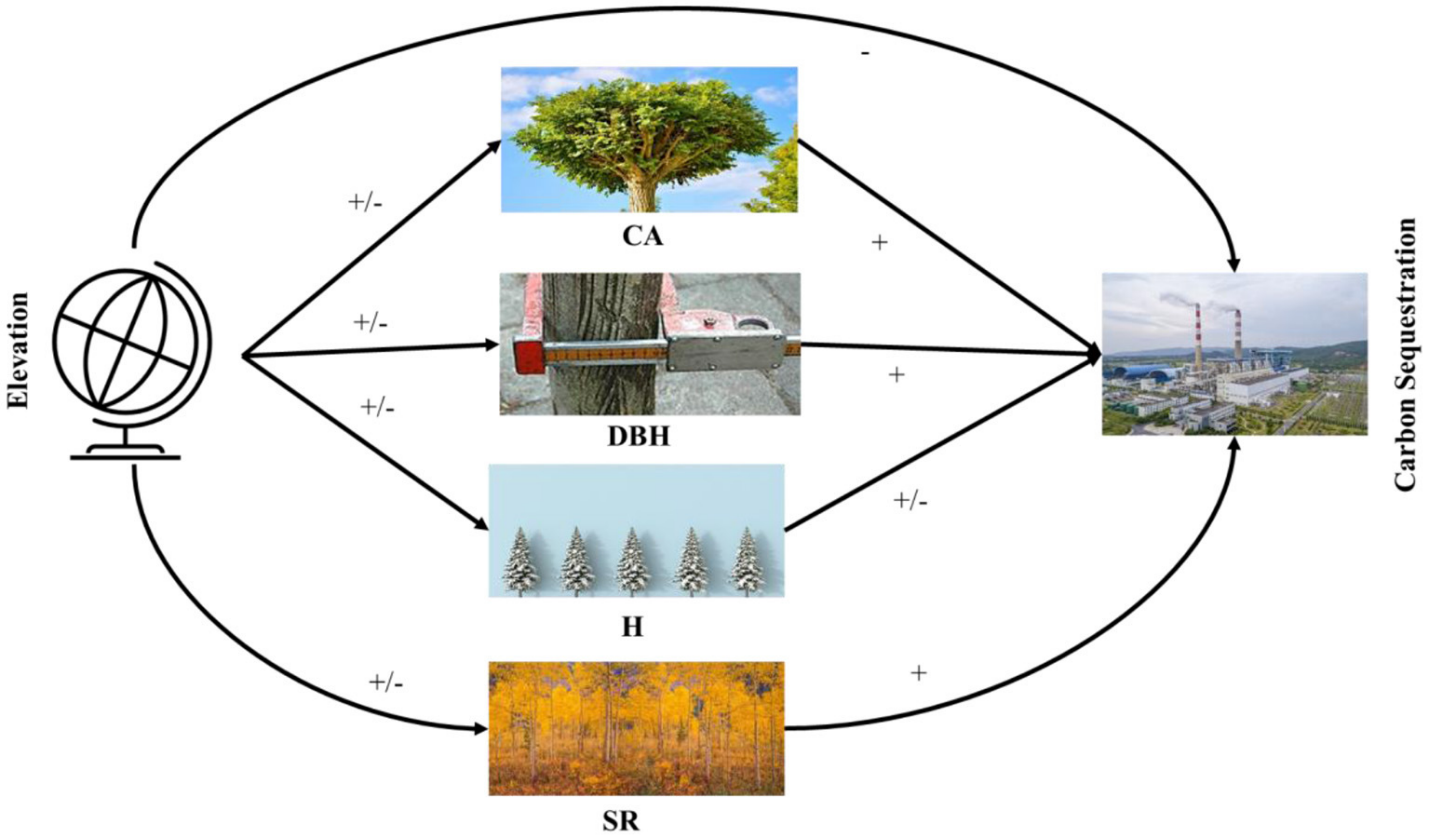
Abstract model explaining the direct and indirect effects of the crown area, DBH, height, species richness, and elevation on carbon sequestration. Hypothesized associations among variables are designated by –, +, or –/+, respectively.

The most critical goal of the current work was to determine the direct and indirect role of CA, H, DBH, and SR on carbon sequestration in the diverse natural forests of Pakistan. Thereby, we also examine the effect of elevation on Carbon sequestration. We hypothesize that carbon sequestration is driven by CA, DBH, H, and SR along the elevation from sea level. We used a structural equation modeling approach to test a hypothesized causal relationship among the response variables using data from 200 plots covering sub-tropical thorn forests, sub-tropical broad-leaved forests, moist temperate mix forests, dry temperate conifer forests, and dry temperate *Pinus gerardiana* (*Chilgoza)* forest plots in Pakistan. The critical research questions are as follows. (i) How does elevation gradients, CA, DBH, H, and SR explain variation in carbon sequestration? We hypothesized that carbon sequestration, CA, DBH, H, and SR decreases along with an increase in elevation (34). (ii) How does carbon sequestration change along the elevation under the influence of explanatory variables CA, DBH, H, and SR? We also hypothesized that tree CA increases carbon sequestration. (iii) How does SR affect carbon sequestration along CA, DBH, H, and elevation? We hypothesized that SR and carbon sequestration have no significant effect on each other. (iv) How does DBH coupled with other explanatory variables, explain variations in carbon sequestration? We hypothesized that DBH has a direct impact on carbon sequestration. (v) How does H impact carbon sequestration along with the CA, DBH, SR, and elevation? We hypothesized there is no effect of H on carbon sequestration (Figure 1).

## 2. Materials and methodology

### 2.1. Study area

**Figure 2:**
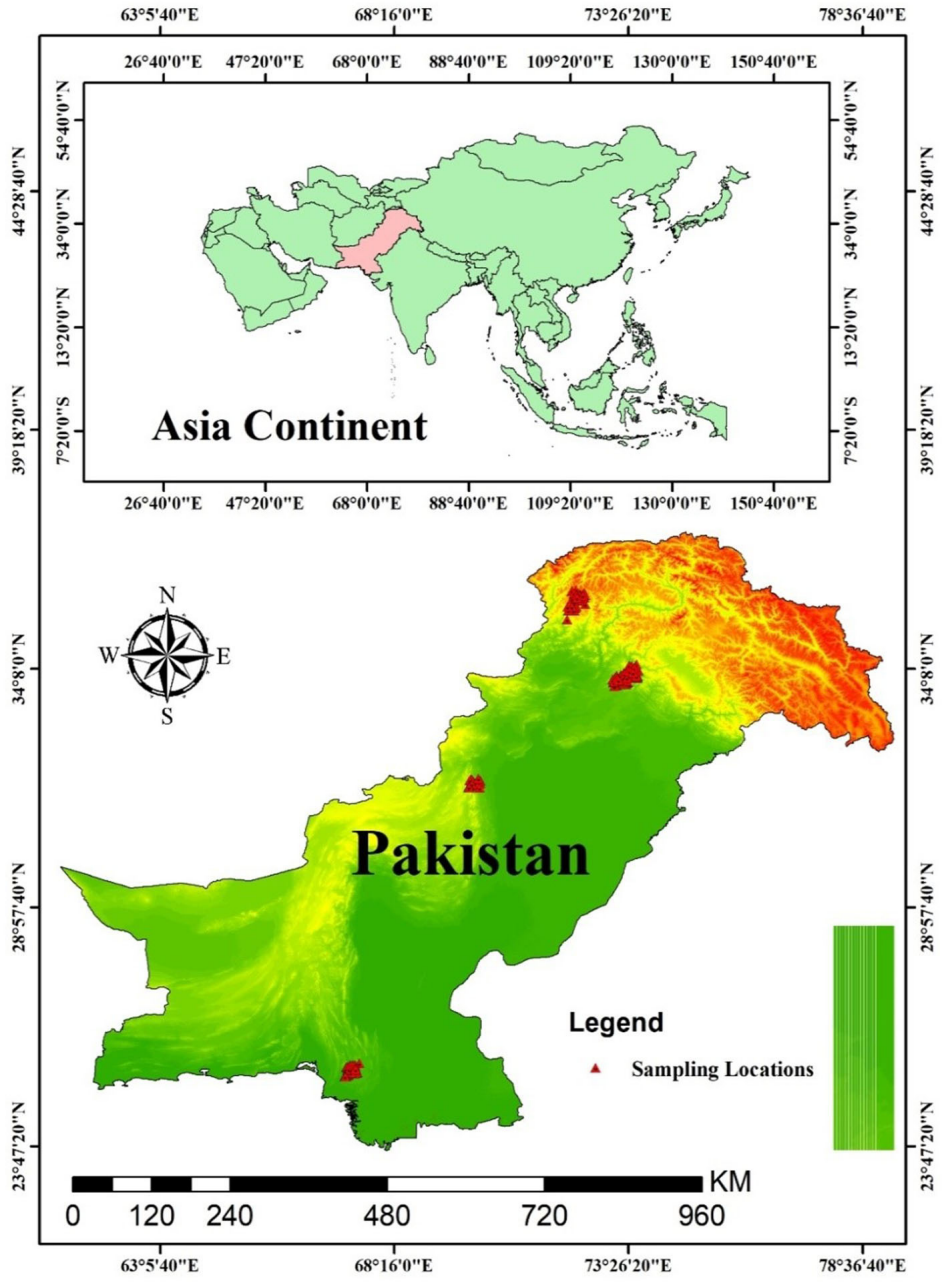
GIS-generated map of the studied forests across Pakistan.

This study covered a geographical area from “25°18.057” to “35°10108” north latitude and “067°11.298” to “071°56.568” east longitude in the different forests of Pakistan. It has an evaluation range between 50 and 2,700 m above sea level. The different forest types studied are the (i) sub-tropical thorn forests (STTF) of Kirthar National Park, Kirthar mountains, Sindh, (ii) sub-tropical broad-leaved forests (STBLF) of Margalla Hills, lower Himalayas, (iii) moist temperate mixed forests (MTMF) of Murree western Himalayas, (iv) dry temperate coniferous forests (DTCF) of Kumrat, Hindu Kush mountains, and (v) the dry temperate *Pinus gerardiana* (*Chilgoza)* forests (DTPGF) of Shirani, Kohe Sulaiman Mountain, Balochistan (Table 1, Figure 2).

**Table 1:** Studied forest types along with their elevation range, mean annual temperature and precipitation.

**S.No**	**Forest types**	**Elevationfrom Sea level (in meters)**	**Mean annual temperature (°C)**	**Mean annual precipitation (mm)**	**Pictorial view**
i.	Sub-tropical thorn forest	56–302	33.8	245.3	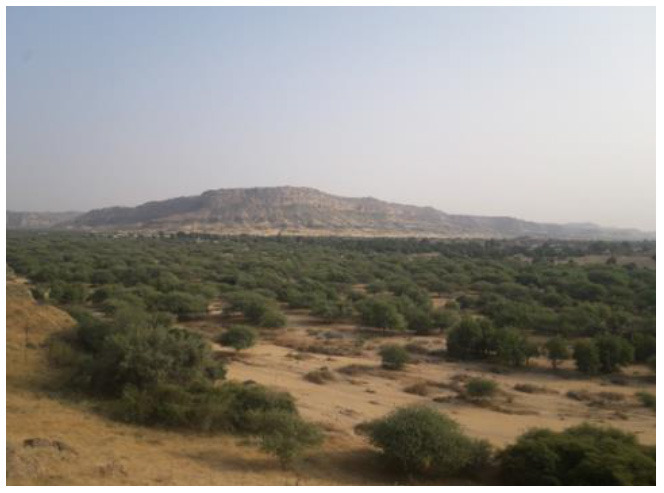
ii.	Sub-tropical broad-leaved forest	555–1,117	27.8	1,572.1	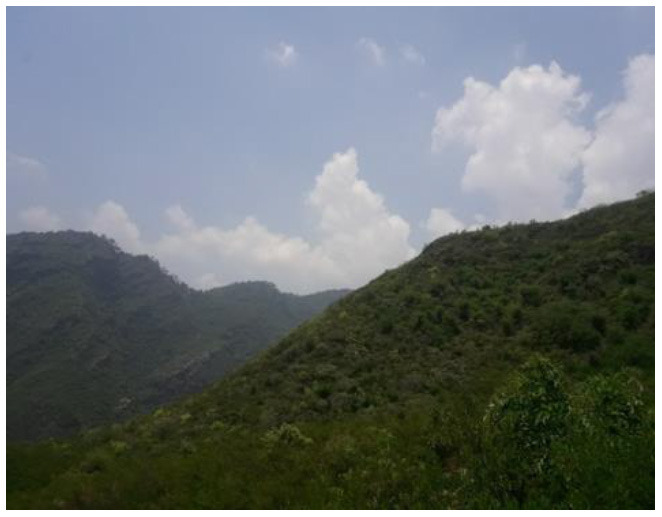
iii.	Moist temperate mix forest	1,249–2,892	17.8	1,596.1	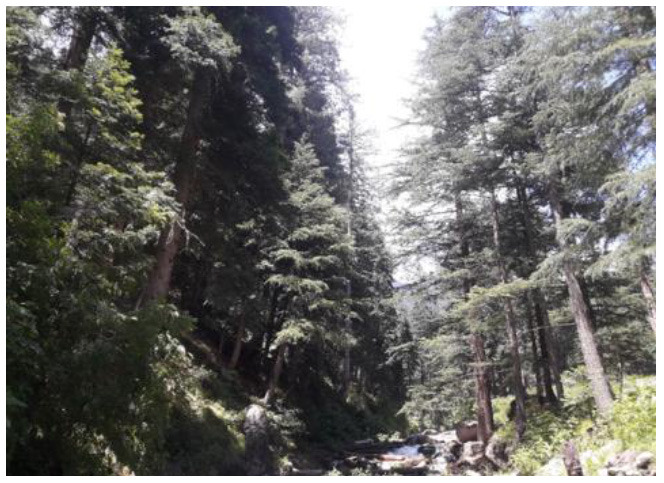
iv.	Dry temperate conifer forest	1,040–2,566	23.4	1,371.8	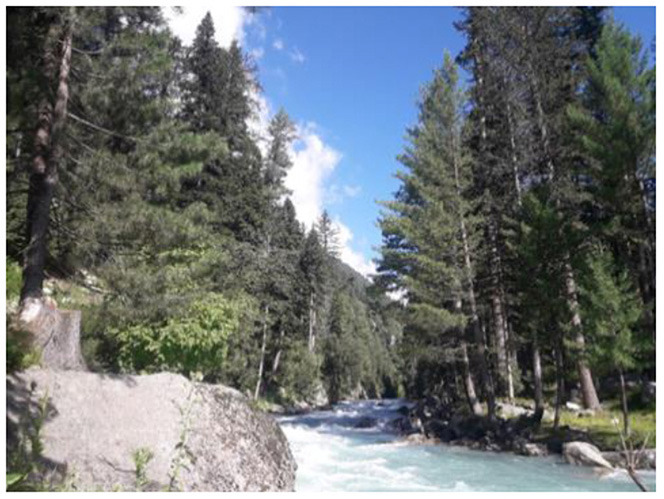
v.	Dry temperate *Pinus gerardiana* (*Chilgoza)* forest	1,841–2,282	25.9	299.0	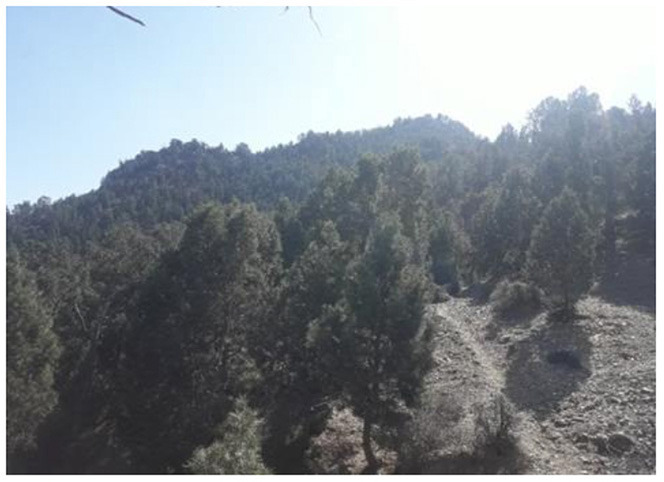

### 2.2. Data collection using quantitative ecological techniques

A detailed description of tools and techniques is explained in this section.

#### 2.2.1. Estimation of carbon sequestrated by plants annually

Carbon sequestration for each woody tree species was calculated through total DBH and total tree height for all the individuals in each plot (35).

#### 2.2.2. Determining total tree green weight

The following algorithmic equation was applied for weighting the carbon sequestration of the woody plant species for 10 years.

(1) $$\begin{array}{l} {\text{W}}_{\text{ag}}=0.15\;\times \;{\text{D}}^{2}\text{H} \end{array}$$

where W_ag_ is the above-ground weight of tree species in pounds (lbs), D is the diameter of the tree trunk in inches (for trees with D>1), and H is the height of the tree in feet. The green weight is the live tree weight. First, we calculated the above-ground green weight using the following method (36).

The belowground system weight of a tree is 20% greater than the above-ground weight. We calculated the total green weight of the tree by multiplying the above-ground weight by 1.2.

(2) $$\begin{array}{l} {\text{W}}_{\text{tgw}}=1.2\times {\text{W}}_{\text{ag}} \end{array}$$

The tree's total average mass is ~72.5% dry matter, and the moisture content of the tree is 27.5%. Hence, we calculate the tree's dry weight by multiplying the total green weight of the tree by 0.725 (37).

(3) $$\begin{array}{l} {\text{W}}_{\text{Dw}}=0.725\times {\text{W}}_{\text{tgw}} \end{array}$$

The average carbon content in the tree is generally 50% of the total tree volume (16, 37). Thus, we determined the weight of carbon in the tree by multiplying the tree's dry weight by 50% or 0.5.

(4) $$\begin{array}{l} {\text{W}}_{\text{c}}=0.5\times {\text{W}}_{\text{Dw}} \end{array}$$

The chemical composition of CO_2_ is one molecule of carbon and two molecules of oxygen. The carbon atomic weight is 12.001115 and the oxygen atomic weight is 15.9994. Therefore, the determination of CO2 weight in trees is the ratio of CO2 C+2 × O = 43.999915 C is 43.999915/12.001115 = 3.6663. Thus, we determined the weight of sequestered carbon dioxide in the tree by multiplying the tree's carbon weight by 3.6663, rounded to 3.67 (16, 38).

(5) $$\begin{array}{l} {\text{W}}_{\text{co}2}=3.67\times {\text{W}}_{\text{c}}\;\left(v\right) \end{array}$$

Tree crown area (CA) was calculated using the formula for the area of an ellipse (Ae):

(6) $$\begin{array}{l} \text{Ae}=\pi \left(0.5x\right)\times \left(0.5y\right) \end{array}$$

where is the crown length and (y) is the width measured perpendicularly (5, 39).

### 2.3. Statistical analyses

A structural equation model (SEM) was constructed based on the following conceptual hypothesis: (a) the direct effect of CA, DBH, SR, H, and elevation on the carbon sequestration (b) the direct effect of elevation on CA, DBH, H, and SR; and (c) the indirect effect of elevation, DBH, CA, H, and SR on carbon sequestration. The goodness of fit was calculated using the goodness of fit index (GFI), the chi-squared test, standardized root mean square residual (SRMSR), the comparative fit index (CFI), Akaike's Information Criterion (AIC), and the Bayesian information criterion (BIC) (40). The causal effect amongst the dependent, independent, and mediator variables was tested in the theoretical model while calculating the predicted variable's total effect, direct and indirect, on a response variable. The description of the variable used in the SEM model is represented in Table 2. A VIF test was carried out to check the multicollinearity in data. In our case, the VIF test's importance is around 2.50. Hence, we proceeded with further regression.

**Table 2:** Description of the independent and dependent variables.

**Variables**		**Description**
H	Height of trees	The height was measured through Clinometer (in Feet).
DBH	Diameter at breast height	The diameter at breast height was measured through DBH tap (in inches).
CA	Crown area	The crown Area was measured by following a standard protocol (in meters).
SR	Species richness	The total number of spices per plot was counted (plot size 20 × 20 m).
E	Elevation	The elevation was noted through GPS.
CS	Carbon sequestration	The amount of carbon sequestration was evaluated through the weight of carbon dioxide in the selected plot is equal to 3.67 times the weight of carbon (16).

The linear regression for each conceptual path was calculated for the complete path measurement of the SEM result, which is shown in Figure 1. However, due to the direct and indirect effects of the other response variables, the results of the bivariate associations may or may not be reliable with the SEM outcome (41–44). Thus, nine series of multiple linear regression models with various response variables were used, for example, (a) the multiple effects of carbon sequestration, CA, H, DBH, and SR; (b) multiple effects of mediator and carbon sequestration; (c) multiple direct and indirect effects of carbon sequestration and response variables. The Hmisc and corrplot packages evaluated the Pearson correlation coefficient matrix.

Before statistical analysis, for normality and linearity, all continuous numerical variables were normalized and standardized and all statistical analysis was done in R 4.0.2 (45).

### 2.4. Empirical model

We determined the conceptual model with several empirical models. Our generalized model as

(7) $$\begin{array}{l} {\text{Y}}_{\text{co}2\text{i}}=\alpha +\beta_{\text{CAi}}+\lambda_{\text{DBHi}}+\delta_{\text{Hi}}+{⋎}_{\text{SRi}}+\theta_{\text{Elevi}}+\mu_{\text{i}} \end{array}$$

where CO_2_ showed carbon sequestration, CA represents aerial expansion, DBH is the diameter at breast height, H indicates the tree height, and Elev is the elevation of the studied areas, where (i) indicates the number of the plots and α, β, λ, ⋎, δ, and θ are the coefficients of the response variables. In contrast, μ is an unobserved variation or error term of the model. We arranged the generalized model into six parts to find the direct, indirect, and total effects and measure all the below equations simultaneously.

(8) $$\begin{array}{l} {\text{Y}}_{\text{co}2\text{i}}=\alpha +\beta_{\text{CAi}}+\lambda_{\text{DBHi}}+\delta_{\text{Hi}}{⋎}_{\text{SRi}}+\theta_{\text{Elevi}}+\varepsilon i \end{array}$$

(9) $$\begin{array}{l} {\text{Y}}_{\text{co}2\text{i}}=\alpha +\beta_{\text{CAi}}+\lambda_{\text{DBHi}}+\delta_{\text{Hi}}+{⋎}_{\text{SRi}}+\varepsilon i \end{array}$$

(10) $$\begin{array}{l} {\text{Y}}_{\text{co}2\text{i}}=\alpha +\beta_{\text{CAi}}+\lambda_{\text{DBHi}}+{⋎}_{\text{SRi}}+\varepsilon i \end{array}$$

(11) $$\begin{array}{l} {\text{Y}}_{\text{co}2\text{i}}=\alpha +\beta_{\text{CAi}}+\lambda_{\text{DBHi}}+\varepsilon i \end{array}$$

(12) $$\begin{array}{l} {\text{Y}}_{\text{co}2\text{i}}=\alpha +\beta_{\text{CAi}}+\varepsilon i \end{array}$$

## 3. Results

**Figure 3:**
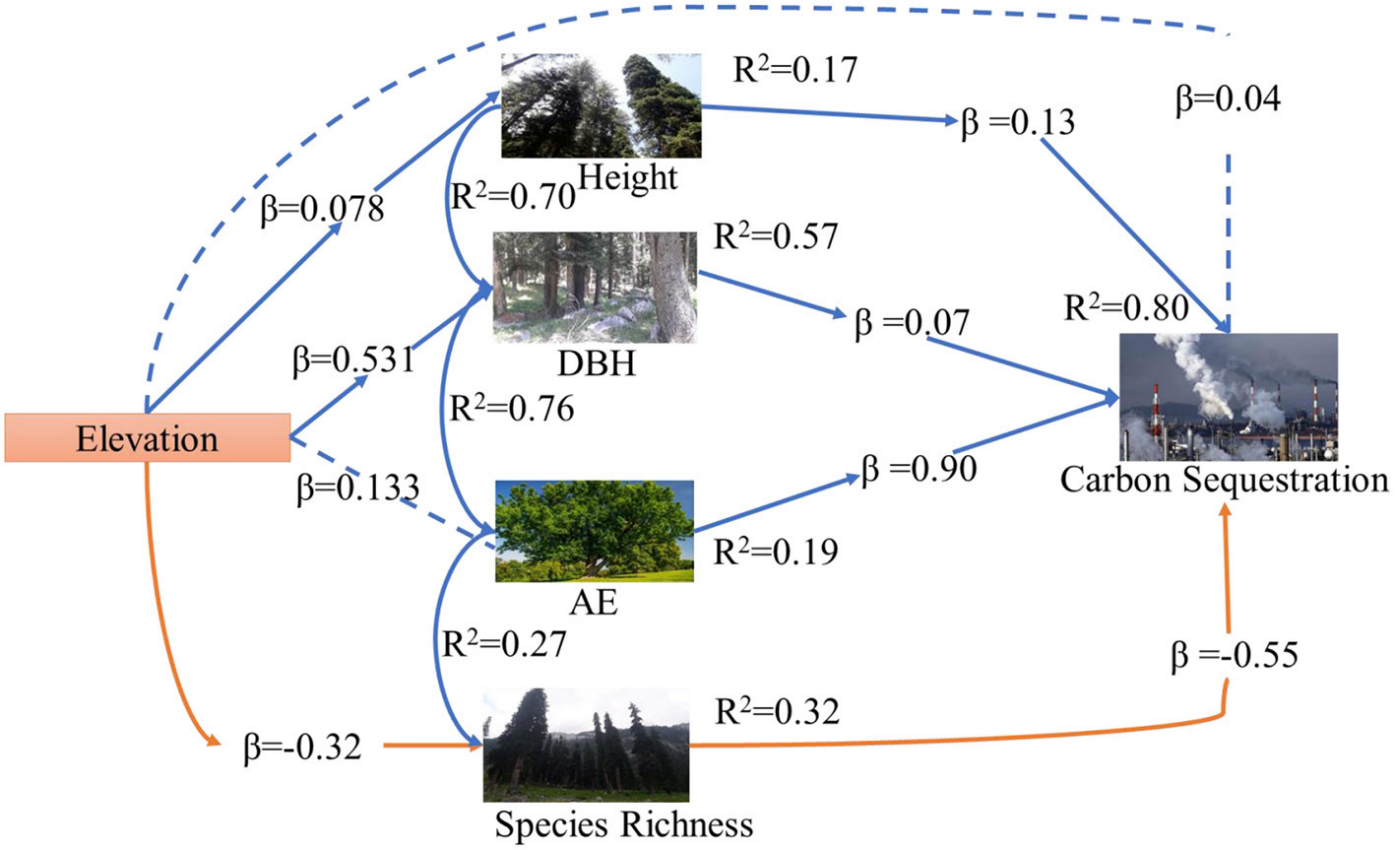
SEM linked elevation, CS, CA, DBH, H, and SR in Pakistan's different forests.

**Figure 4:**
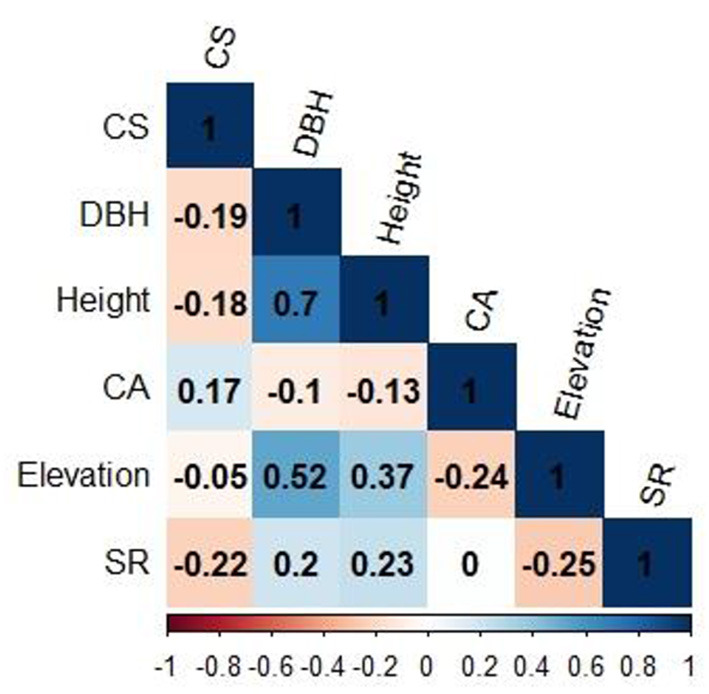
Pearson correlation among all predictor variables. CS, Carbon sequestration; DBH, Diameter at breast height; CA, Crown area; SR, Species richness.

**Figure 5:**
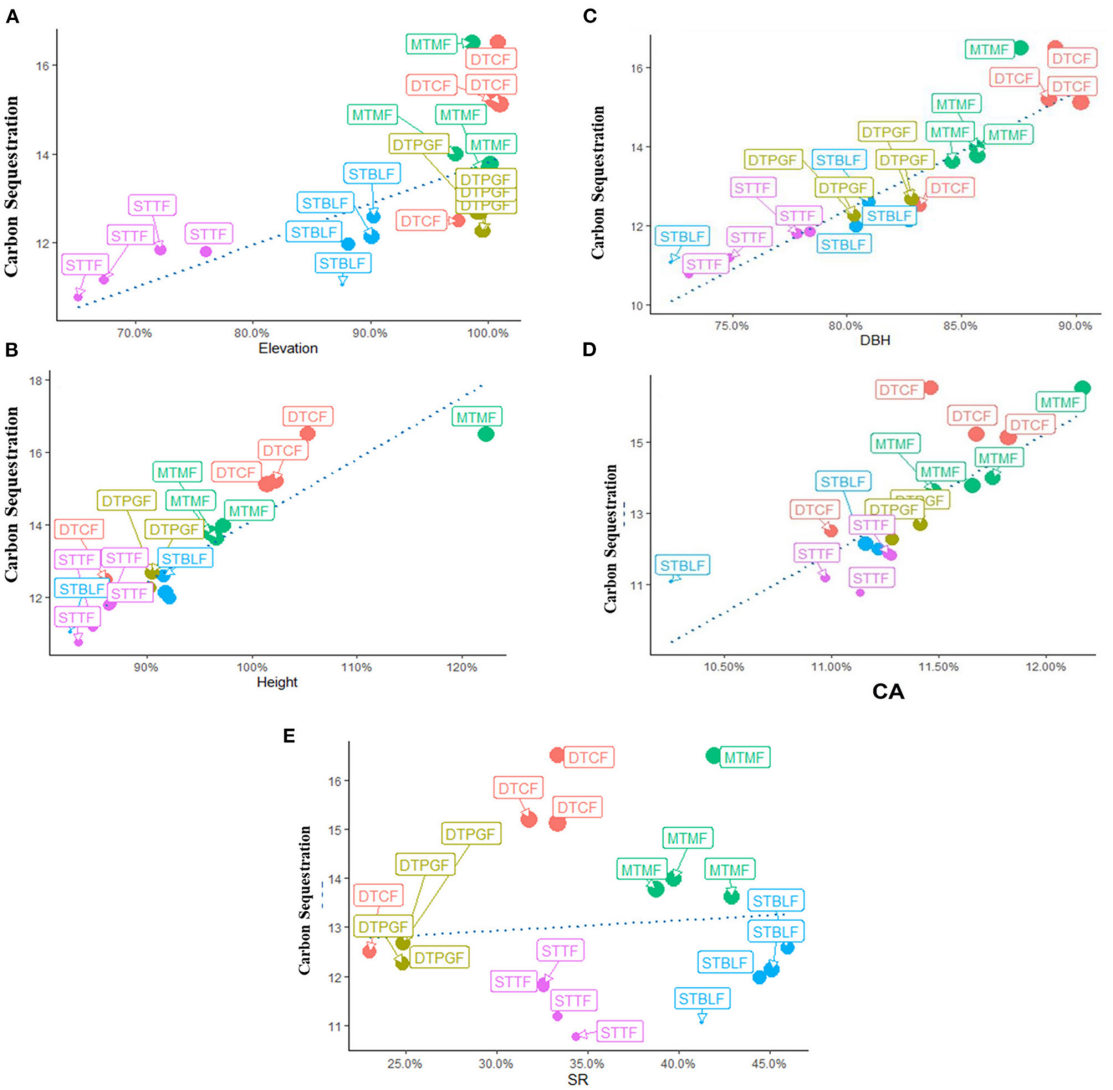
Correlation between the dependent and independent variables, i.e., **(A)** Carbon sequestration with elevation, **(B)** Carbon sequestration with H, **(C)** Carbon sequestration with DBH, **(D)** Carbon sequestration with CA, **(E)** Carbon sequestration with SR. STTF, Sub-tropical thorn forests; STBLF, Sub-tropical broad-leaved forests; MTMF, Moist temperate mix forests; DTCF, Dry temperate conifer forests; DTPGF, Dry temperate *Pinus gerardiana* (*Chilgoza)* forests.

Above and belowground CS was found highest in dry temperate conifer forests (DTCF) i.e., 52.67%, followed by moist temperate mix forests (MTMF) sub-tropical broad-leaved forests (STBLF), dry temperate *Pinus gerardiana* (Chilgoza Forest) (DTPGF), and sub-tropical thorn forests (STTF) with 29.99, 11.66, 4.52, and 1.14% contribution, respectively (Table 3). The SEM described 80% of the CS variation i.e., 57, 32, 19, and 17% under the influence of DBH, SR, CA, and H, of the forest trees, respectively (Figure 3). CA had a strong direct effect on the CS (β = 0.90 and *P*-value < 0.001), followed by the effect of H, DBH and SR with the values of β = 0.13 and *P*-value = 0.009, β = 0.07 and *P*-value = 0.039, and β = −0.55 and *P*-value = 0.001, respectively. The individual direct effect of SR on CS had been negative and significant. At the same time, the separate effects of CA, DBH, and H were positive and significant on CS. The remaining 20% of CS variations were indirectly influenced by elevation. This means that elevation affects CS indirectly through CA, DBH, H, and SR with values of β = 0.133, followed by β = 0.531 and *P*-value < 0.001, and *P*-value < 0.166, β = 0.007 and *P*-value < 0.399, and β = −0.32 and *P*-value < 0.001, respectively (Supplementary Table 1). Whereas, the effect of the mediators (CA, DBH, H, and SR) was examined (β = 0.06 and *P*-value < 0.001, followed by (β = −0.28 and *P*-value < 0.001, β = 0.50.6 and *P*-value < 0.000, and β = 0.32 and *P*-value < 0.068), respectively, on CS (Supplementary Table 1). The total effect of all response variables jointly on CS was 44% positive and significant (Supplementary Table 1). Pearson correlation analysis shows the relative contribution of CS, elevation, CA, DBH, H, and SR (Figures 4, 5). The correlation analyses evaluated the relationship between dependent and independent variables. The analyses show that elevation had a positive correlation with CS. This means that with an increase in elevation up to a limit, carbon sequestration also increases. The relation between carbon sequestration and the biotic variables were positively correlated with each other except SR, ie., when CA, DBH, and H increase the potential for CS also increases (Figure 5).

**Table 3:** Primary result of the study in different forest types.

**S. no**	**Site**	**No of plots**	**No of trees**	**Av. No of trees per plot**	**DBH (%)**	**Height (%)**	**Crown area (%)**	**Species richness (%)**	**Above and belowground carbon sequestration (%)**
1	STTF	40	5,058	126	9.28	7.315	24.52	9.18	1.14
2	STBLF	40	1,923	48	19.15	20.80	23.38	47.95	11.66
3	MTMF	40	5,264	131	27.12	30.00	15.70	31.63	29.99
4	DTCF	40	1,686	42	29.04	30.45	25.18	10.20	52.67
5	DTPGF	40	1,328	33	15.38	11.41	20.20	1.02	4.52

STTF, sub-tropical thorn forests; STBLF, sub-tropical broad-leaved forests; MTMF, moist temperate mix forests; DTCF, dry temperate conifer forests; DTPGF, dry temperate *Pinus gerardiana* (*Chilgoza)* forests.

The direct theoretical paths are examined with each box to remove the difficulties of the regression path in the model (Supplementary Tables 1, 2). The significant standardized regression coefficients are shown in bold text and the insignificant standardized coefficients are shown in the normal text. Model fit statistic: comparative fit index =0.9000; goodness of fit index = 0.9100; standardized root mean square residual = 0.0032; chi sq. value = 10.07 and *P*-value = 0.122; AIC = 2215; CIF = 0.885 and GIF = 0.965, respectively in our model. Supplementary Table 1 shows the standardized direct, indirect, and total SR (b4^*^a1) on various elevation gradients along the regional scale. The indirect effect of CA, DBH, H, and SR significantly affected CS, and the total effect of all variables is also positive and significant (Supplementary Table 1).

The Pearson correlation coefficient measures the strength of the association between the two variables. In the first step of the Pearson correlation, we examined the relationship between two continuous variables and checked the significant effect among all the response variables through the correlation *P*-value. All the variables had a substantial relationship in our case, so we proceeded with further statistical analysis.

## 4. Discussion

This is the complete estimation of carbon sequestration in different forest types, i.e., sub-tropical thorn forest, sub-tropical broad-leaved forest, moist temperate mix forest, dry temperate conifer forest, and dry temperate *Pinus gerardiana* (Chilgoza) forest along regional-scale elevation gradients in Pakistan. It indicates that CA had a substantial direct effect on carbon sequestration, followed by the impact of H, DBH, elevation, and SR, respectively. We will link our findings on carbon sequestration with previous local, regional, and global studies in this section. The findings of carbon sequestration along elevation gradients with the previous study conducted at Mt Changbai, based on H, DBH, and SR, all decreased significantly with an increase in elevation. As a result, elevation negatively affects carbon sequestration (46, 47). Similar to other previous regional level studies indicate that carbon sequestration, all above-ground and below-ground living biomass were significantly and negatively related to elevation on Mt Changbai, probably due to the temperature drop along the elevation gradient, a comparative shortage of water, and low soil temperature for tree growth on high elevation (48). More importantly, this may be because the carbon dioxide did not reach the higher elevations and the trees absorbed more carbon dioxide at the lower elevations. Many researchers have reported that carbon sequestration decreased with increasing elevation (49). Growth can be restricted by water shortages, exposure, reduced temperatures, transpiration rates, deprived soil quality, and low soil temperatures (50). However, the findings vary with studies from other tropical forests, where the biomass decreases with elevation, resulting in a decline in carbon sequestration (22). Xu et al. (51) studied subtropical forests and found that the canopy density is the major biotic factor in modulating vegetation carbon sequestration in forest ecosystems (51).

Ecologists are interested in the potential functional relation between carbon sequestration and species richness (52). The relationship between CS and species richness is still uncertain and depends on other factors, i.e., elevation and physiographic factors. In the present study, we found that carbon sequestration has a significantly negative relationship with species richness. These findings are supported by other studies (53). Sharma et al. (53), from the Garwal region reported a negative correlation between SR and carbon sequestration. Potter (54) concluded that SR is essential, but not the most critical and appropriate metric in biodiversity and carbon sequestration (54). These findings show similarity with our results, i.e., carbon sequestration has a negative relationship with species richness. However, carbon sequestration was directly affected by tree height and DBH in our study. Another study strongly supports the findings of the current one, i.e., carbon density was directly correlated with tree DBH and height (55).

Similarly, Ali et al. (39), concluded that carbon sequestration in the forest is mainly driven by tree height, DBH, crown area, and stand density in the reserve forests of Pakistan (39). Nevertheless, other studies reported no correlation between carbon sequestration with species richness and tree height, suggesting that carbon sequestration has no relationship with SR in forest communities. These differences may be due to environmental conditions like precipitation, temperature, etc., which are not present in our study. Despite this, this study directly shows the effect on carbon sequestration by H, DBH, and CA. Similarly, previous studies reveal that tree crown area in complex tropical forests has a strong positive relationship to carbon sequestration across large-scale ecological gradients. These results highlight new perceptions of the tree crown area complementarity mechanism by which the species having a variety of functional traits cause high resource capture and therefore enhance the above-ground biomass (56, 57).

These findings are supported by Afzal and Akhtar (38). According to these results, the DBH and height have a significant relationship with carbon sequestration. Carbon sequestration mainly depends on the H and DBH of the trees (58). These results indicate that CA, DBH, and H, excluding SR, have directly affected carbon sequestration. Tree height and DBH are directly related to carbon sequestration (59). Ali et al. (60) conducted a study in the subtropical forest of eastern China and a skimmia superba broad leaves forest. They revealed that tree height and DBH significantly correlate with carbon sequestration in the subtropical forest (60). Similarly, Moreno et al. (61) conducted a study in southwest Spain and reported similar results to ours that tree height and DBH have significant and positive correlations with tree carbon sequestration capability (61). They concluded that tree height and DBH have a direct relationship with the above-ground biomass and biomass directly related to carbon sequestration (61). Diaz-Balteiro et al. (62) report a similar result from southwest Spain that an increase in tree height and DBH significantly increase carbon sequestration in the forest ecosystems (62). Similarly, the carbon sequestration capacity of tree height and the DBH of the terrestrial vegetation also significantly correlated with each other (63). All these studies strongly support that carbon sequestration mainly depends on tree growth i.e., CA, DBH, H, and SR.

The present study documents the relevant information on live tree CS along an elevation gradient for different forest types. The present study's findings will help us understand the CS pattern in different forest types. This study will also be helpful for researchers to further understand the relationship between CS, CA, DBH, H, and SR along an elevation gradient. Overall, the current research suggested that extensive studies are required to understand the relationship between carbon sequestration and abiotic factors, i.e. soil physicochemical properties, to understand CS's mechanism and natural driver in the wild forest ecosystem. In this study, we did not focus on the abiotic drivers of carbon sequestration, which may have a significant effect on carbon sequestration in the natural forest ecosystems of Pakistan.

## 5. Conclusions

The present study concluded that a higher above and belowground carbon sequestration potential is found in the dry temperate conifer forest followed by moist temperate forest, sub-tropical broad-leaved forest, dry temperate *Pinus gerardiana* (Chilgoza) forest, and sub-tropical thorn forest. The findings of our research show that CA directly affected carbon sequestration, followed by H, DBH, and SR. There was an insignificant and positive effect recorded of elevation on carbon sequestration. The indirect impact of elevation on carbon sequestration through CA, DBH, H, and SR was recorded positively and significantly. It is also concluded that carbon sequestration is mainly affected by elevation. Furthermore, studies are required to evaluate the relationship between carbon sequestration and multiple biotic and abiotic drivers.

## Data availability statement

The original contributions presented in the study are included in the article/Supplementary material, further inquiries can be directed to the corresponding authors.

## Author contributions

SA contributed to the write-up, fieldwork, and experimental work. SK and AR provided overall supervision, and reviewed and edited the final draft. ZA and ZS helped in data analysis and initial manuscript writing. AU contributed to the field and experimental work. SY and HH provided financial support and edited the final draft. All authors contributed to the article and approved the submitted version.
