# Supplementary material for: Carbon sequestration potential of different forest types in Pakistan and its role in regulating services for public health
Source: Front Public Health. 2023 Jan 13;10:1064586. doi: 10.3389/fpubh.2022.1064586 (PMC9881653; doi:10.3389/fpubh.2022.1064586)
Supplement: Supplementary file 1 [file Table_1.docx]

**Supplementary Table 1**: Direct, the indirect and total effect of the measured environmental variables in relation to carbon sequestration.

| No of observation | | | | | 200 |  |  |
| --- | --- | --- | --- | --- | --- | --- | --- |
| Chi sq. | | | | | 110.0 |  |  |
| P-value | | | | | 0. 72 |  |  |
| R square value | | | | | 0.74 |  |  |
| AIC | | | | | 1851.755 |  |  |
| BIC | | | | | 1907.230 |  |  |
| SRMR | | | | | 0.082 |  |  |
| **Response** | **Predictor** | **β-value** | **S.E** | | **Z-value** | | **P-value** |
| CS | Elev | 0.04 | | 0.035 | | 1.378 | 0.168 |
| **CS** | **SR** | **-0.55***** | | **0.039** | | **3.909** | **0.001** |
| **CS** | **CA** | **0.90***** | | **0.033** | | **27.88** | **0.001** |
| **CS** | **DBH** | **0.07*** | | **0.042** | | **1.819** | **0.039** |
| **CS** | **H** | **0.13**** | | **0.052** | | **2.631** | **0.009** |
| Elev | H | 0.078 | | 0.083 | | 0.936 | 0.349 |
| **Elev** | **DBH** | **0.531***** | | **0.112** | | **4.75** | **0.000** |
| Elev | CA | 0.133 | | 0.096 | | 1.385 | 0.166 |
| **Elev** | **SR** | **-0.32***** | | **0.052** | | **-6.147** | **0.001** |
| **H** | **DBH** | **0.783***** | | **0.027** | | **28.649** | **0.001** |
| **DBH** | **CA** | **0.838***** | | **0.021** | | **39.95** | **0.001** |
| **CA** | **SR** | **0.34***** | | **0.061** | | **5.593** | **0.001** |
| CS.indir1 | b1*a1 | 0.007 | | 0.009 | | 0.843 | 0.399 |
| CS.indir2 | b5*a1 | 0.074* | | 0.039 | | 1.923 | 0.054 |
| CS.indir3 | b6*a1 | 0.032* | | 0.018 | | 1.825 | 0.068 |
| CS.indir4 | b7*a1 | 0.032 | | 0.018 | | 1.825 | 0.068 |
| **H.indir1** | **b5*a4** | **0.506***** | | **0.068** | | **7.475** | **0.001** |
| **DBH.indir2** | **b6*a3** | **-0.28***** | | **0.058** | | **-4.848** | **0.001** |
| **CA.indir3** | **b7*a2** | **-0.06***** | | **0.018** | | **-3.422** | **0.001** |
| SR.indir4 | b4*a1 | 0.032 | | 0.018 | | 1.825 | 0.068 |
| **Total CS** | **a1+b1*a2+b7*a3+b3*a4+b2*a5+b3** | **0.44***** | | **0.14** | | **3.149** | **0.002** |

CS = Carbon Sequestration, Elev= Elevation, H= Height, DBH= Diameter at Breast Height, CA= Crown, SR= Species Richness, AIC = Akaike’s Information Criterion, BIC= Bayesian Information Criterion, SRMSR= Standardized Root Mean Squared Residual.
