# Supplementary material for: Carbon sequestration potential of different forest types in Pakistan and its role in regulating services for public health
Source: Front Public Health. 2023 Jan 13;10:1064586. doi: 10.3389/fpubh.2022.1064586 (PMC9881653; doi:10.3389/fpubh.2022.1064586)
Supplement: Supplementary file 2 [file Table_2.docx]

**Supplementary Table 2:** Summary of multiple linear regression.

| No of observation | | | | 200 | |  |  |  |
| --- | --- | --- | --- | --- | --- | --- | --- | --- |
| Chi sq. | | | | 101.540 | |  |  |  |
| P value | | | | 0. 122 | |  |  |  |
| R square value | | | | 0.80 | |  |  |  |
| AIC | | | | 2215.755 | |  |  |  |
| BIC | | | | 2265.230 | |  |  |  |
| SRMR | | | | 0.082 | |  |  |  |
| Dependant variable Carbon Sequestration | | |  | | | |  | |
| Independent variables |  | β-value |  | SE |  | Z-value |  | P-value |
| **CA** |  | **0.90***** |  | **0.033** |  | **27.88** |  | **0.001** |
| **SR** |  | **-0.55***** |  | **0.039** |  | **3.909** |  | **0.001** |
| Elev |  | 0.04 |  | 0.035 |  | 1.378 |  | 0.168 |
| **H** |  | **0.13**** |  | **0.052** |  | **2.631** |  | **0.009** |
| **DBH** |  | **0.07**** |  | **0.042** |  | **1.819** |  | **0.039** |
| *** p value < 0.000, ** p value < 0.05 and * p value < 0.1 | | | | | | | | |

Elev= Elevation, H= Height, DBH= Diameter at Breast Height, CA= Crown Area, SR= Species Richness, AIC = Akaike's information criterion, BIC= Bayesian information Criterion, SRMSR= Standardized Root Mean Squared Residual.
